# Advancing understanding of maternal age: correlating epigenetic clocks in blood and myometrium

**DOI:** 10.1186/s43682-022-00010-0

**Published:** 2022-05-23

**Authors:** Elise N. Erickson, Anna K. Knight, Alicia K. Smith, Leslie Myatt

**Affiliations:** 1 Midwifery Division, School of Nursing, Oregon Health and Science University, 3455 SW US Veterans Hospital Rd, Portland, OR 97239, USA.; 2 Gynecology & Obstetrics, Emory University, 100 Woodruff Circle, Atlanta, GA 30322, USA.; 3 Obstetrics & Gynecology, School of Medicine, Oregon Health and Science University, 3030 S. Moody Avenue, Suite 110, Portland, OR 97201, USA.

**Keywords:** Epigenome, Genetics, Maternal morbidity, Obstetrics, Parturition, Myometrium

## Abstract

**Background:**

Advanced maternal age is currently a term defined by chronological age. However, a group of biomarkers known as epigenetic clocks, which can predict morbidity and mortality, has been used to estimate measures of biological aging. Uterine myometrial function during the process of parturition may be influenced by aging, as labor dystocia, unplanned intrapartum cesarean birth, and postpartum hemorrhage are more common in older individuals. The purpose of this study was to evaluate the use of epigenetic clocks in maternal myometrium and blood for predicting age and to evaluate the correlation of epigenetic age between the tissues.

**Results:**

We compared epigenetic age in blood and myometrial samples provided by women undergoing planned cesarean birth at term gestation. Chronological age ranged from 20 to 50 with a median (IQR) age of 35.5(8) years. The MethylationEPIC BeadChip was used to obtain DNA methylation data, and then epigenetic age was calculated using the Horvath, Hannum, GrimAge, and PhenoAge clocks. Spearman correlations of epigenetic age with chronological age were calculated. We tested the relationship of epigenetic age in maternal blood to epigenetic age in myometrium. Age acceleration, for each clock, was also correlated between tissues. Twenty-seven participants provided samples, and 21 matched specimens were included in the final analysis after quality control. Spearman correlation between maternal chronological age and epigenetic age were significant in three of the four clocks (pan-tissue Horvath, Hannum, and GrimAge), for both myometrium and blood samples. Correlations between blood epigenetic age and maternal age ranged from 0.72 to 0.87 (all *p* < 0.001). Correlations between myometrial epigenetic age and maternal age were also significant (0.62–0.70, *p* = 0.002), though lower than correlations seen in blood. Maternal blood epigenetic age also correlated with epigenetic age in myometrium with each of these three clocks 0.60 (*p* = 0.004, Horvath), 0.63 (*p* = 0.003, Hannum), and 0.80 (*p* < 0.001, GrimAge). GrimAge age acceleration had the highest correlation between tissues among the clocks (0.49, *p* = 0.02).

**Conclusions:**

Given the limited sample, this study provides insight into the potential use of epigenetic age derived from blood as a proxy for myometrial epigenetic age, which may be a useful biomarker in estimating myometrial biological age in relationship to myometrial dysfunction. GrimAge outperformed the other tested clocks in terms of concordance of epigenetic age and age acceleration between tissues; however, the Horvath and Hannum clocks may be useful depending on the outcome of interest in pregnancy.

## Background

Advanced maternal age (AMA) is a designation based on an expectant mother having a chronological age of 35 or older. AMA status has been associated in population studies with childbirth-related morbidity [1–3]. While many factors influence the physiology of the birth process, the function of the uterine myometrium plays a critical role in labor onset, progress, and for third-stage labor/postpartum involution [4–7]. Labor dysfunction leading to unplanned cesarean and/or postpartum hemorrhage is more common in older individuals [1, 2, 8–10]. Some researchers have documented age-related changes in myometrial function; however, some discrepancies have been reported [11]. Associations include altered arterial impedance [12], myocyte cytoarchitecture [13], myometrial contractility [14], and response to oxytocin stimulation [15] in both human samples and animal models [16, 17]. However, dysfunctional labor, poor myometrial contractility, and hemorrhage also occur in younger populations and conversely many women aged 35 or older do not experience birth-related difficulties, suggesting that factors beyond maternal chronological age determine myometrial function [18].

Currently, determining the likelihood for adverse childbirth-related morbidity linked to myometrial function is not possible prior to labor from any available biomarker measurements or clinical metrics. Aside from prior history of labor dysfunction or a rare uterine anomaly (bicornuate uterus, etc.), clinicians have no tools to predict normal or abnormal parturition until labor begins. Developments in the understanding of biological aging, including epigenetic aging, have proved powerful predictors of morbidity and mortality in other adult populations and disease contexts, more so than chronological age or other previously used measures of biological age (i.e., telomere length). Epigenetic age is a molecular quantification of biological age, which may reflect underlying cellular senescence associated with aging or may be a consequence of the aging process itself [19, 20].

Epigenetic age is calculated based on DNA methylation, which is tissue-specific and changes reproducibly over time. Epigenetic clocks are used to estimate of a biological measure of age based on a weighted average of groups of CpG sites and have been developed for a range of tissues and applications. Among the first clocks developed, the Horvath (Pan-Tissue) clock was validated on a large number of different tissue types, including some endometrial and cervical specimens, which may make it particularly relevant to myometrial function [21]. Next, the Hannum clock was created from a smaller set of CpG sites from whole blood in adults [22]. Both the Horvath and Hannum clocks were trained on chronological age, rather than being developed from survival data. The PhenoAge clock was validated against clinical parameters indicating physiologic dysregulation (*albumin, creatinine, glucose, C-reactive protein, lymphocytes, mean red cell volume, red cell distribution width, alkaline phosphatase)* which was not specific to pregnancy but may be relevant to aging parturient women who are also more likely to suffer metabolic and cardiovascular diseases both before and during pregnancy [23]. Finally, the GrimAge clock reflects a methylation pattern that is closely associated with smoking pack-years and levels of 7 plasma proteins that are associated with disease development/likelihood of death (*adrenomedullin, beta-2-microglobulim, cystatin C, growth differentiation factor 15, Leptin, plasminogen activator inhibitor-1, and tissue inhibitor metallopoteinases-1*) [24].

In addition to estimating biological age, the difference between biological age and chronological age can be informative as the speed at which these age-related changes occur varies from person to person. People experiencing more rapid aging have a biological age that is higher than their chronological age, which is termed age acceleration (AgeAccel). AgeAccel has previously been associated with all-cause mortality or the development of illness among several different clock methods [24, 25]. People with a lower biological age than their chronological age are experiencing age deceleration.

Variability in epigenetic age or AgeAccel has not been studied in myometrial tissue directly and may provide insight into biological aging and function/dysfunction in this key tissue during pregnancy. Few researchers have measured epigenetic age during pregnancy specifically, though a pilot study showed that epigenetic age (Horvath Pan-Tissue and GrimAge) of blood from 35 participants was older during mid-pregnancy when compared to 1 year postpartum [26]. Another similar study examined whole blood samples from 77 women in the second trimester, finding that maternal accelerated epigenetic age (using GrimAge) predicted a shorter gestational age at birth and lower birthweight in the newborn [27], implying AgeAccel may be useful for understanding pregnancy health or fetal growth.

As myometrium during pregnancy can only be accessed during surgical birth, the aim of this study was to determine if existing epigenetic clocks are associated with maternal age in both the myometrium and maternal blood (i.e., validity of the measures). Second, we sought to assess the association between epigenetic age and AgeAccel, measured in maternal blood and myometrium, to determine if blood epigenetic age and AgeAccel can serve as a proxy for myometrial epigenetic age and AgeAccel.

## Results

The final study sample after quality control (*n* = 21) consisted of non-Hispanic, European/white (95%), multiparous individuals (77%) with a median (interquartile range, IQR) chronological age of 35.5 (8) years, mean (SD) gestational age at delivery of 38.7 (0.8) weeks and mean (standard deviation, SD) pre-pregnancy body mass index of 27.8 (7.8) kg/m^2^. Infant birth weight (grams) had a mean (SD) of 3192 (483). First, we calculated epigenetic age for each clock and compared it with chronological age. The median (IQR) blood epigenetic age for the pan-Tissue Horvath clock of 39.2 (8.8) years was significantly higher than chronological age (*p* = 0.008). Similarly, the median epigenetic age (Horvath) for myometrium was also higher at 49.4 (4.2) years (*p* < 0.001). Interestingly, Horvath’s pan-tissue clock estimated all myometrial specimens to be of age 40 or greater despite the chronological age of the sample spanning ages 20–50. The Hannum and GrimAge-derived epigenetic age estimates were significantly younger than chronological age for blood (Hannum = 28.5 (8.0) years, GrimAge = 28.2 (6.8) years, *p* < 0.001) and for myometrium (Hannum = 19.7 (5.0) years, GrimAge = 32.7 (6.9) years, *p* = 0.002).

We then examined the correlation between epigenetic age and chronological age for each tissue for the various clocks. Using a Spearman rho (*ρ*) of at least 0.5 as a benchmark (considered a moderate correlation [28]), we found that the Horvath, Hannum, and GrimAge clocks met criteria with significant correlations of epigenetic age with chronological age in both tissues (Table 1). However, the PhenoAge epigenetic clock was not correlated with chronologic age in myometrial tissue (*ρ* = 0.3, *p* = 0.15); thus, we focused on analyses on epigenetic age and AgeAccel for the other three clocks.

**Fig. 1:**
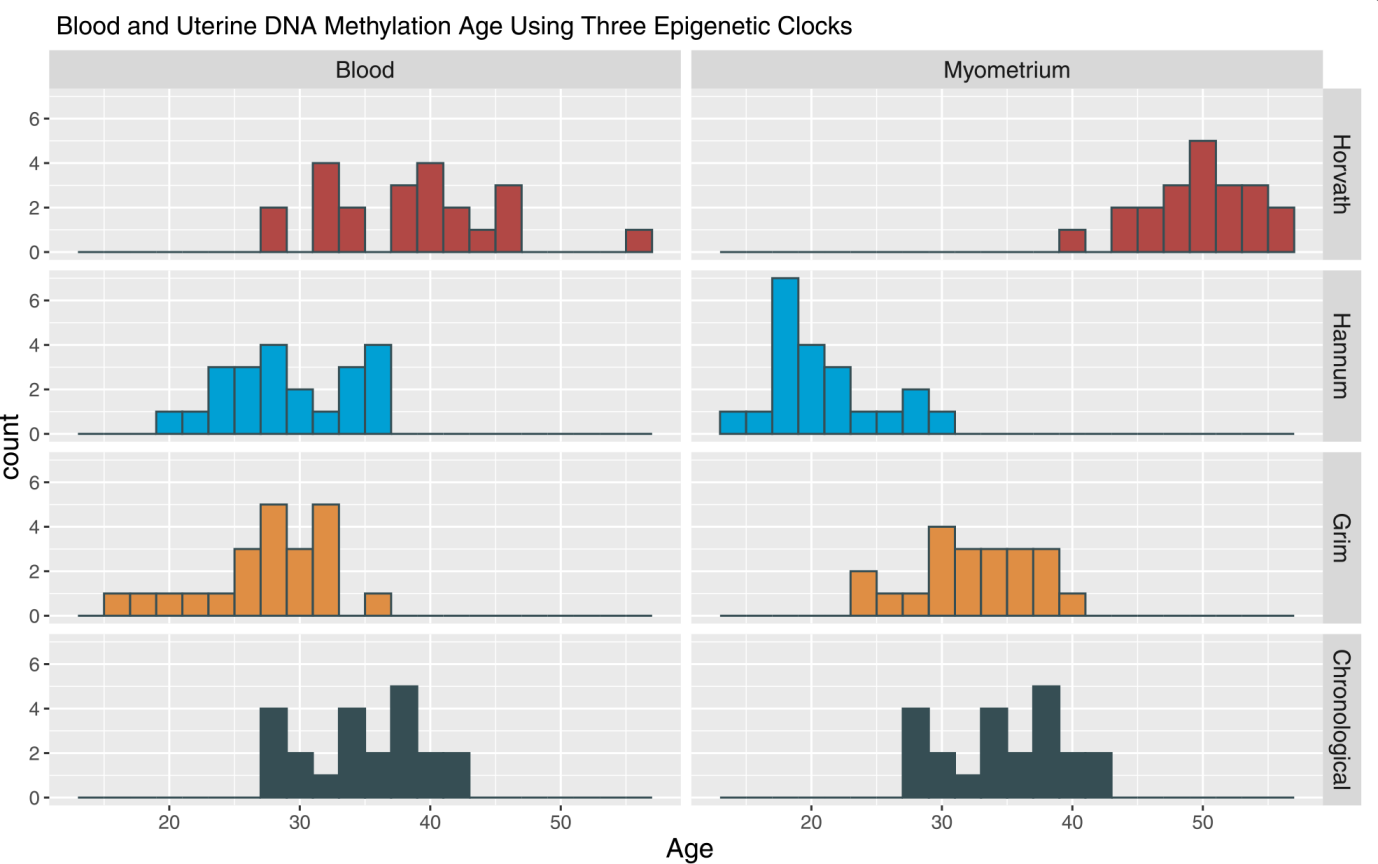
Blood and myometrial DNA methylation using three epigenetic clocks. The distribution of epigenetic age for each clock (labeled on right hand side of graph) by tissue is presented with the distribution of chronological age of the sample for reference

Epigenetic age derived from the blood and myometrium is shown in Fig. 1.

**Fig. 2:**
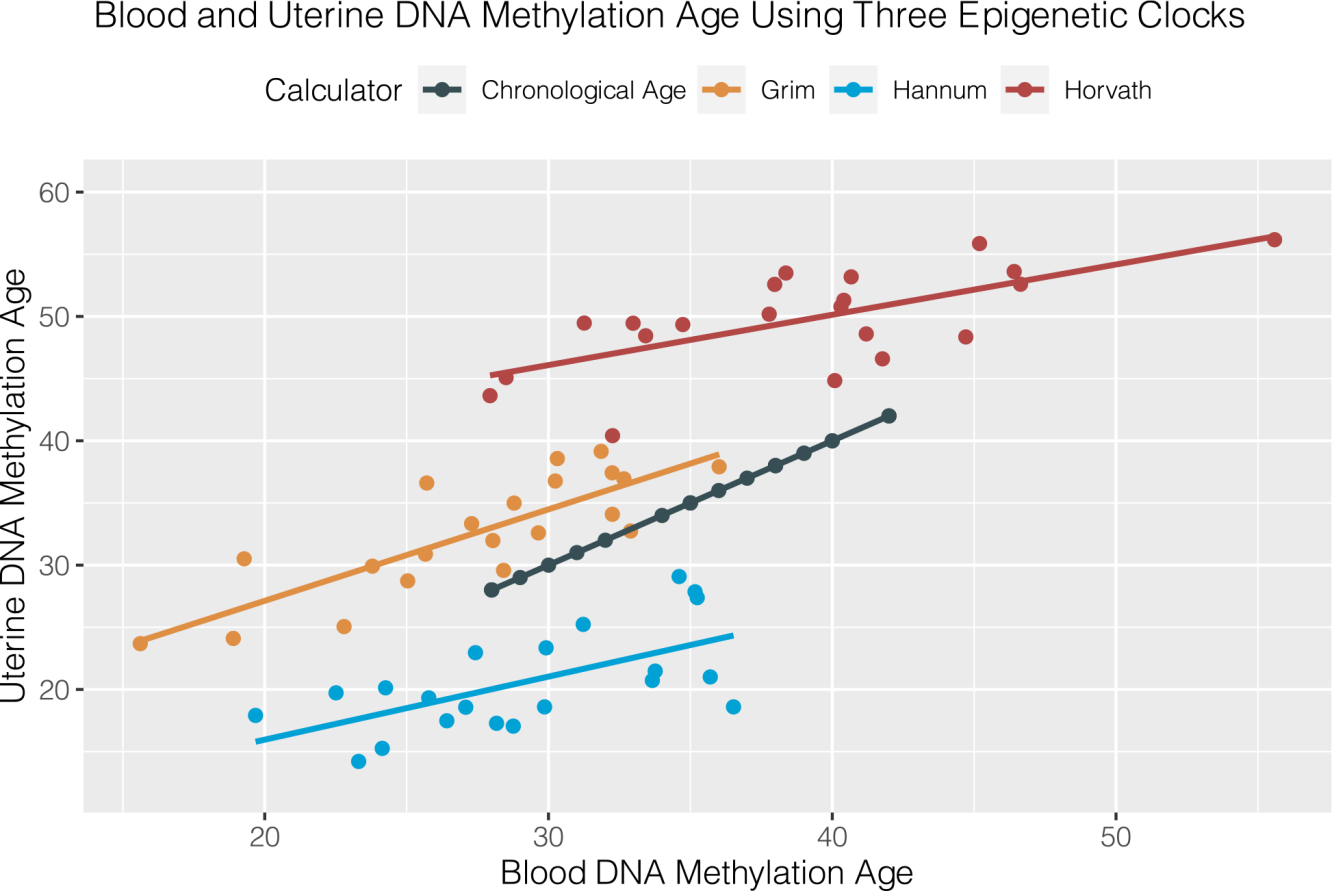
Correlation between blood and myometrial DNA methylation age. Spearman *ρ* and significance for each clock as follows: Horvath (0.60, *p* = 0.004), Hannum (0.62, *p* = 0.003), and GrimAge (0.81, *p* < 0.001)

Correlations between epigenetic age in blood and epigenetic age in myometrium for the Horvath, Hannum, and GrimAge clocks are noted in Table 2 and Fig. 2. The GrimAge calculator had the highest correlation coefficient (*ρ)* of 0.81 (*p* < 0.001); however, the Horvath and Hannum clocks had moderate correlations of *ρ* = 0.62 and *ρ* = 0.60, also both significant (*p* = 0.004 and *p* = 0.003 respectively).

Then, we calculated AgeAccel. The correlations between age acceleration between the two tissues shown in Table 2. Notably, GrimAge Accel between the two tissues had the highest correlation of the clocks we tested *ρ* of 0.49 (*p* = 0.02).

We examined maternal pre-pregnancy body mass index (BMI) in relationship to blood epigenetic age derived from each clock as a post-hoc examination; however, no correlations were statistically significant. Myometrial AgeAccel (GrimAge) was moderately correlated with BMI, *ρ* = 0.52 (*p* = 0.01). This association could be spurious and needs testing in larger samples in future work, as after applying Bonferroni correction for multiple testing, the association was not significant.

## Discussion

In this study, we examined the epigenetic age of uterine myometrium sampled from term pregnant individuals undergoing cesarean birth in comparison to maternal chronological age and to epigenetic age of matched maternal blood samples using the Horvath pan-tissue clock, the GrimAge clock, the Hannum clock, and the PhenoAge clock. The primary findings were that (1) three of the four clocks measured epigenetic age within the two tissues and (2) epigenetic age was significantly positively correlated between blood and myometrium in the same three clocks. However, AgeAccel was only correlated using the GrimAge calculator. To our knowledge, this is the first study to report using epigenetic age to assess myometrium and matched blood epigenetic age.

In our study, while epigenetic age was correlated with chronological age each clock’s median biological age significant differed from maternal chronological age (Grim/Hannum younger and pan-tissue Horvath older). This discrepancy between clocks may be a feature of how the clocks were originally generated and validated. Epigenetic age, as described by Horvath et al. [21], was developed as a tool for estimating biological age and derived from elastic net regression that identified a set of CpG sites that are correlated with age among 82 different cell and tissue types (including cervix, endometrium but not myometrium). Therefore, the original Horvath clock is used to examine epigenetic age in multiple tissue types. The original Horvath Clock was followed by several other epigenetic “clocks” which were validated against other useful measures of physiologic function and consist of sets of CpG sites for each clock, with little overlap. Further description of the epigenetic clocks is available.

Pregnancy represents a unique state of change in the body, and may lead to unique changes in DNA methylation patterns (especially in the myometrium and reproductive tissues), therefore, current epigenetic age estimates may not be as useful because they were validated in non-pregnant populations. This may explain why we had different biological age estimates from the different clocks used in our study. This finding also creates an opportunity for future validation of a pregnancy-specific maternal epigenetic clock that examines aging across the pregnancy and reflects biological age related to pregnancy-specific outcomes. Alternatively, cellular aging of different organs/tissues may occur at different time points over the life course, as found in mouse models of aging [29]. Furthermore, researchers using Horvath’s clock have shown that human breast tissue epigenetic age seems to be older than age derived from blood DNA, similar to our findings comparing myometrium/blood [30]. However, this difference could be related to less precise age estimates for hormone responsive tissue like breast/endometrium using the CpGs included in the original Horvath clock [21]. Work is ongoing to improve the breast-tissue specific CpG patterns estimating epigenetic age [31].

The study of biological aging using an epigenetic clock is a relatively new approach yet has not been robustly applied to studying/predicting poor maternal health outcomes. One study of endometrial (uterine lining) aging also found that epigenetic age in the endometrium was relatively older than chronological age [32], similar to our finding using Horvath’s clock in myometrium. While endometrium and myometrium share some similarities including embryonic origin (mesenchyme) and steroid hormone-responsiveness, the myometrial layer develops distinct features of smooth muscle through induction by the epithelial layer (future endometrium) [33, 34]. Uterine endometrium proceeds to proliferate and degenerate with cyclic changes in ovarian hormones. The stroma retains clonogenic properties of mesenchymal stem cells [35, 36]. Cyclic modulation of endometrium may be controlled in part by epigenetic mechanisms [37]. With aging, the function of endometrium is affected by the loss of stem cells [38] as well as by decreased proliferation necessary for establishing implantation, driven by lower estrogen production.

In contrast, research on myometrium in healthy reproductive aged humans is more limited. While myometrium does not undergo significant changes with the ovarian cycle, there is evidence of stem cell involvement in tissue function for remodeling during pregnancy and possibly for coordinating myometrial contractions during labor [39, 40]. In addition, myocytes respond to rising steroid hormones during pregnancy with progesterone repressing gene expression for contraction-associated proteins during early pregnancy. As estriol rises, myocytes increase gene transcription for contraction-associated proteins (oxytocin receptors, prostaglandin receptors, and gap junctions) [4, 41]. Some prior studies did not find differences in contractile ability in myometrial tissue specimen by maternal age [11, 15]; however, others have noted lower responsiveness to oxytocin stimulation [42] or lower electromyographic activity [43]. Clinical studies report that older parturients have longer labors [44] and more often need oxytocin in labor [8], and higher dosages of oxytocin are needed for third-stage labor to prevent uterine atony [45]. The specific reasons for the differences observed by studies of vitro contractility studies and clinical outcomes by age prompts further study.

Other studies of epigenetic aging involving pregnancy have largely focused on newborn or child epigenetic clock measures or outcomes. For example, maternal depression (as well as SSRI use) has been associated with epigenetic age differences in newborn DNA [46–49]. Researchers might consider if maternal aging is associated with mental illness or other measures of social stress during pregnancy as has been noted in non-pregnant samples [50, 51]. Relevant to preterm birth, GrimAge AgeAccel assessed prenatally predicted shorter gestational age and lower birthweight in the newborn [27]. Epigenetic age has been applied to the study of health disparities by race/ethnic subgroups [52, 53]. Given that disparities in maternal mortality and morbidity by racial groups widens with advancing age [18, 54], examining epigenetic age would also be useful for those interested in examining biological age in association with experiences of stress, discrimination, economic, or social disadvantage [55, 56]. Given the rising age for first and subsequent pregnancies (as well as age-related disease like cardiovascular disease and diabetes), the concept of biological aging and epigenetic age specifically may be a useful measure for future research on maternal morbidity or development of age-related disorders in pregnancy.

Furthermore, given our finding of higher GrimAge AgeAccel in myometrial tissue with higher body mass index, future work should consider both age and body composition on uterine aging and outcomes. While maternal age is closely linked to more labor dysfunction, a growing scientific literature also links higher body mass to poor outcomes as well [7, 57]. Maternal adiposity has been linked to alterations in placental DNA methylation and gene transcription; however, epigenetic clock methods were not applied to the methylation data in this study [58]. Whether BMI plays a role in accelerated aging, which mediates the relationship between age and myometrial function, should be investigated further.

### Research implications

Myometrial function has been studied in relationship to the aging reproductive population. This study contributes to the body of work examining how age-related biological differences may be important to myometrial function during pregnancy. From a broad perspective, further work on epigenetic age may highlight important variability in outcomes across the maternal age spectrum. If consistent with other literature on epigenetic aging, this tool could be an informative indicator of “Advanced Maternal Age” in terms of biological age as advancements in epigenetic assays occur (i.e., improved speed, lower cost). However, before that is possible, the significance of these epigenetic age differences (AgeAccel) needs to be determined in future studies of physiologic function and clinical outcomes. For example, will Horvath’s clock or GrimAge be a more useful predictor of healthy physiologic labor and birth or severe maternal morbidity? Our study helps support the premise that blood is a useful proxy for myometrial epigenetic age, given future studies of outcomes of vaginal birth will need to rely upon the blood derived DNA for epigenetic age assessment. Samples obtained earlier in pregnancy might could be useful in studying birth-related outcomes linked to myometrial function (i.e., prolonged pregnancy, prolonged labor etc.) but may also be informative at predicting other pregnancy-related conditions like preeclampsia. Furthermore, epigenetic age might also be a compelling biomarker for determining if interventions designed to improve the health of pregnant women (diet/exercise, stress reduction, aspirin therapy) are detected in the biological age of the individual.

### Strengths and limitations

Despite a more limited sample size than anticipated, significant correlations in epigenetic age were consistently found between myometrium and blood. Data availability and having a small sample size limited our ability to assess potential confounders that may influence age acceleration. This study would have been strengthened by in vitro studies of myometrial contraction in relationship to aging but was not possible given the preservation methods of the tissue. Furthermore, none of the participants underwent labor; thus, we cannot conclude that these samples represent a wider sample of the population due to the nature of having planned cesarean birth. Limited clinical data and sample size prohibited more stratification by specific obstetric or medical conditions.

## Conclusions

Using existing epigenetic clock methods, epigenetic age in maternal blood is correlated with maternal myometrium at delivery, and GrimAge AgeAccel correlated between tissues as well. Future studies should evaluate the clinical relevance of AgeAccel in predicting age-associated complications during pregnancy and parturition.

## Materials and methods

### Aim

The first aim of this study was to determine if existing epigenetic age measures (clocks) relate to maternal age in both the uterine myometrium and maternal blood (i.e., validity of the measures). Second, we sought to assess the association between epigenetic age measured in maternal blood and myometrium for potential future use of blood epigenetic age as a biomarker of myometrial epigenetic age.

### Design

This study was a comparative study examining epigenetic age, as calculated using different epigenetic clocks, in both maternal blood and myometrium.

### Participants

Data used in this study were available via a pregnancy-related tissue repository managed by (blinded institution). Potential participants engaged in a consent process with trained research staff to participate, which included donating samples of myometrium, whole maternal blood, placenta, and cord blood. Participants whose samples were used in this study also specifically provided consent to participate in genetic research. The pregnancy repository is approved by the research ethics board at (blinded institution). This study accessed the repository after receiving a request for determination that our study did not constitute human subjects research as it was using de-identified data and tissues. Limited clinical information was available in the repository. Participants gave birth at two locations (Oregon Health and Science University (Portland, OR) and Imperial College, University of London: Chelsea and Westminster Hospital). Material data transfer and data use agreements were established between institutions; de-identified samples were collected from (Oregon Health and Science University (Portland, OR) and Imperial College, University of London: Chelsea and Westminster Hospital) and were included in an existing tissue repository at Oregon Health and Science University.

Tissue samples were obtained from pregnant individuals undergoing planned cesarean birth at 37 weeks or greater [59]. Indications for delivery varied, including elective primary or repeat cesarean; several participants had chronic or gestational hypertension or proteinuria though none of the cesarean births were performed urgently due to a medical indication. A small portion of full-thickness myometrium (measuring approximately 2 cm × 2 cm in size) was excised bluntly from the edge of the hysterotomy incision shortly after delivery of the newborn. The surgical staff handed the myometrial specimen to study staff shortly after birth, the myometrium was rinsed briefly in phosphate-buffered saline, dissected from serosa and any endometrium, if present, and flash frozen in liquid nitrogen in small sections. Whole blood was also frozen in aliquots after being collected in standard EDTA tubes in the 1–2 h prior to delivery via venipuncture. Whole blood was frozen and stored at − 80 °C until DNA was isolated. Specimens were collected from a total of 27 participants; however, only 25 had both tissue samples collected.

## Procedures

### DNA extraction and methylation

The DNA was isolated from whole blood specimens and myometrium using QIAmp DNA Mini Kits. DNA was then quantitated by fluorescence using Quant-iT^™^ Pico-Green^®^ dsDNA assay kit (Invitrogen | Thermo Fisher Scientific).

DNA methylation was then assessed using Illumina’s MethylationEPIC BeadChip (Illumina, San Diego, USA). Five hundred nanograms of each sample was bisulfite converted using an EZ DNA Methylation Kit (Zymo), amplified, hybridized, and imaged. DNA methylation data for over 850,000 CpGs was generated per sample and preprocessed using R statistical suite (version 3.6.1). IDAT files from the Illumina array were loaded into the R package, Minfi. Noob background correction was performed, and detection *p*-value and beta value sample files were generated for over 850,000 CpGs. During quality control, 5 samples with low signal, and 2532 CpG sites with high missingness were excluded. Previously identified cross-reactive probes were dropped [60]. After quality control, 819,723 CpG sites and 21 matched myometrial and blood samples remained.

### Epigenetic clock calculator

After completing quality control, epigenetic age was calculated separately for blood and myometrial samples using Horvath’s website (https://dnamage.genetics.ucla.edu). Specifically, using the online calculator, we examined pan-tissue Horvath [21], Hannum [22], PhenoAge [23], and GrimAge [24] clocks for this study.

### Statistical analyses

Epigenetic age data for both myometrium and blood resulting from the four different clock calculators were examined with histograms for dispersion within the tissues. Spearman correlation was used to first compare each epigenetic age to maternal (chronological) age given the distribution. Clocks that correlated significantly with maternal age at rho ≥ 0.5 in both tissues were considered in the subsequent analyses (this included the pan-tissue Horvath, GrimAge, and Hannum clocks). We then examined the Spearman correlation between the tissues types for the valid clocks. Tests of differences in epigenetic age to chronological age by tissue were conducted using paired *t*-tests/Wilcoxon signed-rank tests as appropriate for each clock. For each clock, we calculated the epigenetic age acceleration (AgeAccel) from the residual of a linear regression of DNA methylation age (epigenetic age produced by the given clock) on chronological age creating the following variables: pan-tissue Horvath AgeAccel, GrimAge AgeAccel, and Hannum AgeAccel. For each clock, we then calculated Spearman correlations for AgeAccel between tissues. Finally, we tested the correlation of maternal pre-pregnancy BMI with each clock’s estimated epigenetic age and AgeAccel across the sample as a post hoc examination given the limited clinical data available for these specimens (with Bonferroni correction). The rationale for this additional analysis was based on knowledge that higher maternal BMI has been linked to uterine dysfunction during labor [7] and that researchers have reported associations of higher BMI with greater AgeAccel (using pan-tissue Horvath clock) in both liver and adipose tissue samples [61, 62]. Statistical significance was set at *p* < 0.05.

## Figures and Tables

**Table 1:** Spearman rho correlations of epigenetic age by tissue and between tissue type using matched tissues from pregnant individuals at term (*n* = 21)

Correlation	Epigenetic clock	*ρ*	*p* value

Blood epigenetic age with maternal age	Horvath	0.72	< 0.001
	Hannum	0.87	< 0.001
	PhenoAge	0.73	< 0.001
	GrimAge	0.80	< 0.001
Myometrial epigenetic age with maternal age	Horvath	0.62	0.002
	Hannum	0.63	0.002
	PhenoAge	0.22	0.33
	GrimAge	0.70	< 0.001

**Table 2:** Spearman Rho correlations between tissues for epigenetic age and age acceleration

Correlation	Epigenetic clock	*ρ*	*p* value

Blood and myometrial epigenetic age	Horvath	0.60	0.004
	Hannum	0.62	0.003
	GrimAge	0.81	< 0.001
Blood and myometrial epigenetic age acceleration	Horvath	0.28	0.21
	Hannum	0.20	0.37
	GrimAge	0.49	0.02

## Abbreviations

AMA: Advanced maternal age
AgeAccel: Age acceleration
IQR: Interquartile range
SD: Standard deviation
BMI: Body mass index
